# External Beam Radiation Therapy in Treatment of Malignant Pheochromocytoma and Paraganglioma

**DOI:** 10.3389/fonc.2014.00166

**Published:** 2014-06-27

**Authors:** Jennifer Vogel, Aileen Sia Atanacio, Tamara Prodanov, Baris Ismail Turkbey, Karen Adams, Victoria Martucci, Kevin Camphausen, Antonio Tito Fojo, Karel Pacak, Aradhana Kaushal

**Affiliations:** ^1^NIH, Radiation Oncology Branch, National Cancer Institute, Bethesda, MD, USA; ^2^NIH, Program in Reproductive and Adult Endocrinology, Eunice Kennedy Shriver National Institutes of Child Health and Human Development, Bethesda, MD, USA; ^3^NIH, Molecular Imaging Program, National Cancer Institute, Bethesda, MD, USA; ^4^NIH, Medical Oncology Branch, National Cancer Institute, Bethesda, MD, USA

**Keywords:** radiation, pheochromocytoma, paraganglioma, malignant, neuroendocrine

## Abstract

**Purpose:** Pheochromocytomas (PCCs) are neuroendocrine tumors arising from the adrenal medulla or as paraganglioma (PGL) from extra-adrenal sites. While usually benign, a small fraction is malignant. Multi-modality therapy is used in treating malignant disease; however, little data exist on the role of external beam radiation therapy (EBRT). In this retrospective review, we assessed response to EBRT in malignant PCCs or PGLs.

**Methods and Materials:** Records of patients treated at the National Institutes of Health who received EBRT between 1990 and 2012 were studied. Patients were assessed for symptomatic control, biochemical response, local and distant control by response evaluation criteria in solid tumors v1.1 or stable disease on imaging reports, toxicity by radiation therapy oncology group (RTOG) criteria, and survival.

**Results:** There were 24 patients treated who received EBRT to lesions of the abdomen (*n* = 3), central nervous system (*n* = 4), and bone (*n* = 40). Lesions were treated with 3D conformal EBRT to a mean dose of 31.8 Gy in 3.3 Gy fractions, or fractionated stereotactic radiosurgery to 21.9 Gy in 13.6 Gy fractions. Patients experienced acute (*n* = 15) and late (*n* = 2) RTOG toxicities; no patient experienced acute toxicity ≥4 or late toxicity ≥2. Symptomatic control was achieved in 81.1% of lesions. Stable radiographic response was achieved in 86.7% of lesions with progression in 13%. Distant progression was observed overall in 75% of patients and average survival was 52.4 months.

**Conclusion:** Malignant PCC and PGL often do not respond well to current systemic therapies. In these cases, EBRT can be considered in patients with symptomatic, localized disease progression.

## Introduction

Pheochromocytomas (PCCs) are neuroendocrine tumors arising from chromaffin cells of the adrenal medulla. Closely related tumors of the extra-adrenal paraganglia are termed as paragangliomas (PGLs). Adrenal PCCs are the most common chromaffin tumors, occurring in about 80–85% of cases. The remainder occur as PGLs, which may be classified as branchiomeric, including glomus jugulare and carotid body tumors; intravagal; aorticosympathetic; and visceral-autonomic. These tumors are rare, with an annual reported incidence of two to eight per million population and prevalence in 0.2–0.4% of hypertensive patients. They are characterized by their ability to synthesize, store, and secrete catecholamines although some, especially those of branchiomeric or intravagal origin, are non-functional. Elevated urinary fractionated and plasma free metanephrines have been shown to be sensitive markers of disease and are therefore often used in diagnosis. Although less sensitive for diagnosis, chromogranin A levels are also valuable in following response to therapy and monitoring for recurrence (1).

While most occur as sporadic tumors, about 30–35% is associated with hereditary syndromes. These include multiple endocrine neoplasia type 2 arising from mutations in the rearranged during transfection (*RET*) proto-oncogene; von Hippel–Lindau (VHL) syndrome caused by mutations of the *VHL* gene; and von Recklinghausen’s disease due to mutations of the neurofibromatosis type 1 (*NF1*) gene. In addition, mutations in genes encoding succinate dehydrogenase subunits (*SDHD*, *SDHC*, and *SDHB*) are associated with familial PGL syndromes (PGL1, PGL3, and PGL4), respectively.

Currently, the only diagnostic criterion for malignant disease is the presence of metastasis. The proposed incidence of malignant disease ranges from 3 to 36% with generally lower rates of malignancy in PCC compared to PGL. However, rates of malignancy have been reported as high as 50% in patients with *SDHB* mutation. Overall 5-year survival in the setting of benign disease is between 90 and 95%. Prognosis of malignant PCC and PGL is poor, with a 5-year survival between 34 and 72%. While benign disease can generally be definitively treated with surgical resection, malignant PCC and PGL currently have no cure. Patients are treated with multi-modality therapy, which may include surgical resection, radiopharmaceutical therapy, chemotherapy, and radiofrequency ablation. Within these treatment options for malignant disease, the role of external beam radiation therapy (ERBT) remains largely undefined.

Ascribing a role to EBRT in the treatment of malignant PCC/PGL is difficult given limited numbers of patients and reports in the literature. There have been case reports on malignant PCC/PGL in which EBRT was delivered to unknown total doses. In these cases, irradiation was delivered to the primary tumor or metastatic sites with varying symptomatic control and most often with progression of local and distant disease (2–6). However, these reports may be of limited utility especially in PCC/PGL, given results from a 1978 literature review, which concluded that radiation therapy is beneficial for symptomatic relief of bone and lymph node metastases only at high doses (7).

Use of high-dose EBRT outside of bone and lymph node metastases was associated with significant normal tissue toxicity in older case reports, limiting its utilization for malignant PCC/PGL (8, 9). More recent case reports in which intensity modulated radiation therapy (IMRT) or stereotactic radiosurgery and fractionated stereotactic radiosurgery (SRS/FSRT) are used, however, have allowed delivery of higher doses of radiation without significant normal tissue toxicity, resulting in durable local radiographic and symptomatic control at broader sites of metastatic disease (10). However, all of these reports are based on limited numbers of patients and lesions with the exception of a recent study on the role of EBRT and ^131^I-MIBG in non-head-and-neck PCC or PGL (11) (Table 1). In addition, this is the only study utilizing radiographic and biochemical responses to measure outcomes following EBRT in a subset of the patients reviewed. In the majority of case reports, patient characteristics as well as radiation techniques and outcomes are incompletely documented and direct comparison of the patients across these reports is difficult.

**Table 1:** **Malignant PCC/PGL cases treated to known total dose**.

Reference	*N*	Site	Dose (Gy)	Results
Fishbein et al. (11)	17	Malignant PCC/PGL outside the head and neck	Median 40	76% local control or significant symptomatic relief
Pham et al. (10)	7	Bony metastasis (5) and abdominal primary (2)	Mean 44 ± 0.04	71% effective in relieving pain (5/7)
Yoshida et al. (12)	3	Pelvic, para-aortic, left inguinal lymph nodes	Median 55	Control of catecholamines, hypertension, and swelling
Elder et al. (13)	4	Osseous metastases	Median 20	100% with decreased urine catecholamines and temporary alleviation in pain
Teno et al. (14)	2	Sacral and vertebral metastases	Mean 30	No significant change, exacerbated hypertension
Brodkey (15)	2	Vertebral metastasis	Mean 47	Resolution of myelopathy
Hamilton (16)	4	Bony metastasis	Median 40	Symptomatic improvement (50%); distant progression (100%)
Jindel (17)	2	Bony metastasis	Mean 38.7	Resolution of myelopathy and swelling
Yu (18)	3	Various	Median 25	Higher doses may have resulted in sustained remission
Olson (19)	2	Bony metastasis	Mean 30	Resolution of pain and weakness; distant progression
Siddiqui (20)	3	Abdominal primary, femur, spinal cord	Median 25	Resolution of pain and neurologic symptoms; distant progression
Drasin (7)	2	Vertebral metastasis	Mean 36.5	Resolution of pain, slowed progression
James et al. (21)	10	Various	Median 25	50% treated with <2500 Gy without symptomatic or survival benefit
Scott (8)	15	Various	Median 32.5	Some control of pain and BP, complicated by leukopenia
Holsti (22)	6	Various	Median 44.5	66% survival at 2 years
Moloney (9)	6	Various	Median 30.75	Pain control at bony metastasis; poorly tolerated at primary

The objective of this study was to better characterize the utility of EBRT within multi-modality therapy by performing a retrospective review analyzing symptomatic, radiographic, and biological responses to treatment of patients with malignant PCC or PGL.

## Materials and Methods

A retrospective chart review of endocrinology patients treated at the National Institutes of Health between 1990 and 2012 was performed. Patients were selected if they received EBRT for pathologically confirmed malignant PCC/PGL. Irradiation was performed using either ^60^Co machine or 4- to 23-MV photons from a linear accelerator. Patient charts were assessed for: age, sex, familial syndromes, pre-treatment and post-treatment plasma and urinary metanephrine (PMNs, UMNs), and chromogranin A (CgA) levels within 5–6 months of radiation therapy, total radiation dose and fraction size, radiation technique, symptomatic response, local and distant radiographic response or control, radiation toxicities, additional therapies after treatment, and survival time.

**Figure 1:**
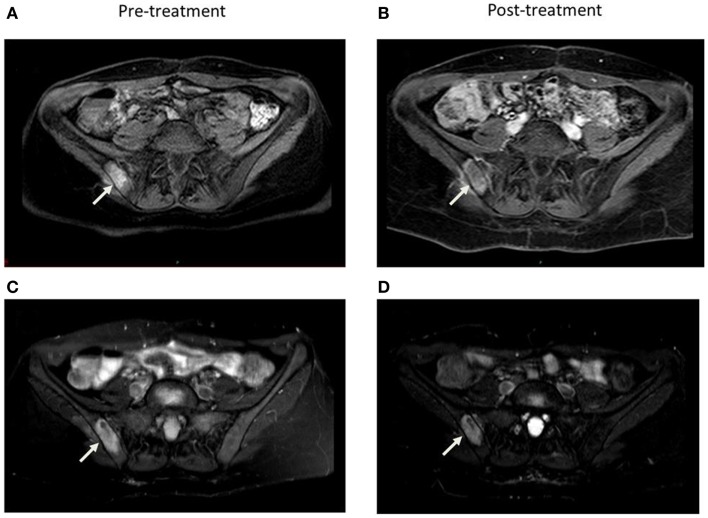
**Comparison of axial 1 month pre and 5 months post-treatment contrast enhanced (A, B) and STIR (C, D) MRI images demonstrate decrease in vascularity and size of the right sacroiliac joint lesion**.

Radiographic response was determined by response evaluation criteria in solid tumors (RECIST) v1.1 based on computed tomography (CT) or magnetic resonance imaging (MRI) reports for a subset of eight patients with 12 lesions with pre and post-treatment imaging available for evaluation (Figure 1). The criteria were applied to these patients for both local and distant, or non-target, disease. For the remainder of patients without imaging available, local and distant control was defined as stable lesion size and progression defined as any increase in size or number as documented by imaging reports. Given the retrospective nature of this study, imaging was performed at variable intervals based on physician discretion. Local and distant control was determined at the time of last available post-treatment imaging. Time to progression (TTP) was defined in months beginning on the first day of EBRT to first progression of local or distant disease in both subsets of patients. Toxicity was graded according to the radiation therapy oncology group (RTOG) acute and late criteria extrapolated from documentation in charts and on-treatment notes. Survival time was defined as months from ERBT to death or to last follow-up.

## Results

Treatment courses for 24 patients with a total of 47 lesions who met these criteria were reviewed. Patients were an average age of 34.1 years at diagnosis (range 12–59 years). Eight patients presented with metastases and 16 recurred with metastases an average of 6.2 years (2–264 months) after resection of the primary tumor. One patient had a *RET* mutation, one had *SDHD* mutation, and nine had *SDHB* mutations. Prior to EBRT, 16 patients underwent surgical resection alone; 3 received resection followed by systemic chemotherapy (CVD, 3–26 cycles); 1 underwent resection with RFA to liver metastases; 1 underwent resection with systemic yttrium-90 therapy; and 1 underwent resection, systemic chemotherapy (CVD, 2 cycles), and RFA to iliac crest lesions. Only two patients received biopsy alone prior to EBRT (Table 2).

**Table 2:** **Patient characteristics**.

Characteristic	
**AGE AT DIAGNOSIS**	
Average	39.5
Range	12–59
**SEX**	
Male	14 (58%)
Female	10 (42%)
**PRESENTATION**	
Metastatic disease	8 (33%)
Local disease	16 (67%)
Average time to metastasis	6.2 years
**MUTATION STATUS**	
SDHB	9 (38%)
SDHD	1 (4%)
RET	1 (4%)
**THERAPY PRIOR TO EBRT**	
CVD	4 (17%)
Resection	22 (92%)
RFA	2 (8%)
**INDICATION FOR RT**	
Pain	32 (68%)
Cord compression	2 (4%)
Tinnitus/hearing loss	2 (4%)
Double vision	1 (2%)
Residual disease	10 (21%)
**SITE TREATED**	
Bony metastases	40 (85%)
Abdominal tumor	3 (6%)
CNS	4 (9%)

Patients received EBRT to abdominal, CNS, and bone lesions. Bone lesions were treated with 3D conformal EBRT to a mean dose of 32.6 Gy in 3.9 Gy fractions, CNS tumors to a mean of 30.3 Gy in 2.1 Gy fractions, and abdominal disease to a mean of 54 Gy in 1.8 Gy fractions. The overall mean was 31.8 Gy in 3.3 Gy fractions. Treatments were delivered by SRS to a single bony lesion to 24 Gy; by SRS to a CNS lesion at a dose of 14 Gy; and by FSRT to two abdominal masses to 24.0 and 25.5 Gy in 8.0 and 8.5 Gy fractions, respectively. Of the 47 sites treated, 37 were symptomatic. Of lesions which were symptomatic, improvement in symptoms was reported in 81.1% (*n* = 30), with response of two symptomatic lesions included in analysis lost to follow-up.

Following RT, 13 patients received systemic chemotherapy for an average of 8.7 cycles (CVD; cytoxan and adriamycin; temozolomide; MS275, 6–18 cycles). Patients were treated with radiopharmaceutical therapy: nine were treated with ^131^I-MIBG therapy, one received radioactive octreotide, and one received radioactive Yttrium-90. Patients received additional surgery (*n* = 8) and RFA (*n* = 2). Only three patients died from progressive disease (PD) and mean follow-up time was 52.4 months.

For lesions evaluated by RECIST response, stable disease (SD) was achieved in 83.3% and PD in 16.7% (*n* = 2) with an average TTP of 22.47 months. For non-target disease, 28.6% of patients (*n* = 2) achieved complete response (CR), 14.2% had non-PR/non-CR (*n* = 1), and 71.4% had PD with an average TTP of 13 months. Of the lesions not evaluable by RECIST, local control was achieved in 82.8% of lesions, with 11.4% progressing (*n* = 4) at an average of 14.8 months. For non-target disease, 16.7% of patients had no distant disease progression while 83.3% progressed at an average of 17.6 months. Overall, local control was observed in 86.7% of patients and progression in 13.3%; distant control observed in 25% of patients and progression in 75% (Table 3).

**Table 3:** **Results of EBRT therapy**.

Patient	Primary	Field site	Dose (cGy)	Fractions	Radiation technique	Outcome	Months follow-up
1	PGL	R iliac fossa	3000	15	3D conformal	Decreased pain. Stable LD, DP at 8 months	40
		L pubic symphysis	3000	15	3D conformal		
2	PCC	C1-C6	3000	10	3D conformal	Pain resolved. Stable LD, DP at 18 months	110
3	PGL	T6-L1	3000	10	3D conformal	Decreased pain. Stable LD, DP at 2.4 months	5.9
4	PGL	L abdomen	4500	25	3D conformal	Decreased pain. Stable LD, DP at 7 months	23
			900	5	3D conformal		
5	PCC	T9-T11	3500	14	3D conformal	Improved pain. Stable LD, DP at 4 months	20
		R femur	2000	5	3D conformal		
6	PCC	L SI joint	3500	14	3D conformal	Continued pain. Stable LD, DP 11.2 months	7
		R femur	3500	14	3D conformal		
7	PGL	R glomus jugulare	1400	10	Gamma Knife	Asymptomatic. Stable LD, distant CR	56.4
8	PGL	R skull base	5400	30	TomoTherapy	Continued symptoms. Stable LD, distant CR	6.7
9	PCC	Thoracic/lumbar spine	4050	29	3D conformal	Decreased pain. Stable LD; DP at 188.1 months	220
10	PGL	C3	2400	1	SRS	Asymptomatic. Stable local/distant disease	39
11	PGl	T3-T7	2000	8	3D conformal	Resolution of symptoms T3-T7; unchanged C6-T2. Stable LD, DP at 9 months	64
		T3-T7	3600	18	IMRT		
		C6-T2	3000	12	3D conformal		
12	PCC	L1-L5	3825	17	3D conformal	Lessened pain. Stable LD, DP at 6 months	51
13		L hip	3000	10	3D conformal	Pain resolved. Stable LD, DP 4.13 months	9.9
		L4	3000	10	3D conformal		
14	PCC	T10	2100	1	IMRT	Improved pain. LP at 24 months, DP at 8 months	33
15	PCC	T6-T12	3000	10	3D conformal	Improved pain; asymptomatic at R/L femur, occipital, and frontal lobes. LP at T6-T12 at 15 months otherwise stable. DP at 2 months.	68
		Sacrum	3000	10	3D conformal		
		C5-T2	3000	10	3D conformal		
		L1-L5	3000	10	3D conformal		
		T3-T5	3000	10	3D conformal		
		R femur	3000	10	3D conformal		
		L femur	3000	10	3D conformal		
		T12	2100	3	IMRT		
		R mandible	3000	10	3D conformal		
		Frontal lobe	3750	15	3D conformal		
		Occipital lobe	3750	15	3D conformal		
16	PCC	Porta hepatis	2550	3	FSRT	Unchanged pain, stable LD, no DP	10
17	PCC	L1, bilateral SI joints	3750	15	3D conformal	Improvement in pain. Progression at L1 at 18 months, DP at 1 month	26
		R hip, femur	3000	10	3D conformal		
		C4-T3	3000	10	3D conformal		
		L hip, femur	3000	10	3D conformal		
		L4-5. Pelvis	1800	9	3D conformal		
18	PCC	Organ of Zuckerkandl	2400	3	FSRT	Asymptomatic or decreased pain. LP at T11 at 3 months, DP at 0.75 month	27
		T11	2800	4	3D conformal		
		T8/T9	2000	5	3D conformal		
19	PCC	Skull base	5000	25	3D conformal	Improved double vision. LP at C7-T12	5
		C7-T12	3750	15	3D conformal		
20	PGL	R iliac wing	5000	10	3D conformal	Asymptomatic. Stable LD, DP 9 months	38
21	PCC	R clavicle	3000	10	3D conformal	Improved pain. Stable LD, no DP	123
		R hip	3000	10	3D conformal		
22	PGL	L glomus jugulare	3200		3D conformal	Improved pain. LP at 40.8 months, DP at 74.1 months	74.1
23	PGL	T1-L2	4500	25	3D conformal	Asymptomatic. Stable LD, DP at 46.5 months	128
24	PCC	Sacrum	3000	15	3D conformal	Improved pain. Stable LD, DP at 50 months	69

Of patients with familial syndromes (*n* = 11), 78.5% of symptomatic lesions improved following radiation therapy. Patients with familial syndromes evaluated by RECIST criteria had 84.6% local SD with 15.3% local PD (*n* = 2) at an average of 13.43 months TTP. They had 92.3% distant PD with 7.6% distant SD and an average TTP of 25.85 months. Of those not evaluated by RECIST criteria, 75% had local control and 25% (*n* = 1) local progression at 28.63 months TTP. Distant CR was achieved in 33.3% (*n* = 1) and distant progression occurred in 66.7% with an average TTP of 42.7 months.

Patients who received 3D conformal or IMRT responded best symptomatically at a higher average dose of 33.7 Gy while average dose for patients without improvement was 31.7 Gy (*P* = 0.39). Local SD was achieved at an average of 34.75 Gy compared to 31 Gy for those with PD (*P* = 0.15). Progressive distant disease was observed at an average dose of 33.3 Gy with stable distant disease observed at an average of 36.0 Gy (*P* = 0.34). In our study, 100% of patients treated with SRS/FSRT achieved local control (*n* = 4) compared to 85.3% (*n* = 35) of those treated with standard fractionation.

All 24 patients were evaluated for biochemical response by chromogranin A, free plasma metanephrines, and fractionated urine metanephrines. Six patients had chromogranin A levels before and after radiation; four of these showed a positive biochemical response. Six patients had free plasma metanephrine levels, three with a decrease after radiation. Four patients had fractionated urine metanephrine levels, three with a decrease in value after radiation therapy (Table 4).

**Table 4:** **Biochemical responses to EBRT**.

Patient	Biochemical test	Pre-EBRT measurement	Post-EBRT measurement
2	PMN (nl: 12–61 pg/mL)	132	207
6	Chg A (nl: 0–225 ng/mL)	296	257
7	Chg A (nl: 0–225 ng/mL)	278	540
	PMN (nl: 12–61 pg/mL)	32	36
14	Chg A (nl: 0–225 ng/mL)	2160	1780
	UMN (nl: 44–261 μg/24 h)	73	89
19	Chg A (nl: 0–225 ng/mL)	23,500	10,900
	UMN (nl: 44–261 μg/24 h)	96	89
	PMN (nl: 12–61 pg/mL)	14	27
20	UMN (nl: 44–261 μg/24 h)	48	110
	PMN (nl: 12–61 pg/mL)	25	23
22	Chg A (nl: 0–225 ng/mL)	93	84
	UMN (nl: 44–261 μg/24 h)	93	110
	PMN (nl: 12–61 pg/mL)	51	18
23	Chg A (nl: 0–225 ng/mL)	65,000	137,000

Toxicities from treatment were both acute and late. Patients experienced RTOG grade 1 acute skin (*n* = 3), esophageal (*n* = 4), and upper gastrointestinal (*n* = 2) toxicity, as well as grade 2 upper gastrointestinal (*n* = 4), and grade 3 upper gastrointestinal (*n* = 1) and esophageal (*n* = 1) toxicities. There were two reported late toxicities, one esophageal which subsequently resolved and one spinal cord which was permanent in one patient treated by SRS to C3 to 2400 cGy. No patient suffered RTOG acute toxicity grade ≥4 or any RTOG late toxicity grade ≥2.

## Discussion

In this retrospective analysis, we evaluated patients with malignant PCC/PGL with primary lesions in the head, neck, and abdomen. Our patients had metastatic lesions to bones, to the abdomen, and to the CNS. This series is the largest on the response of PCC/PGL to EBRT and, unlike many previous case series and case reports, utilizes newer technologies of 3D conformal radiation therapy including IMRT as well as SRS/FSRT. In addition, a subset of our patients was evaluated using standardized radiographic criteria and assessed for biochemical responses to localized radiation therapy. We found an overall symptomatic improvement in 81.1% of patients after radiation therapy regardless of site or radiation technique, with overall local control of 86.7% in patients treated to mean doses of 31.8 Gy in 3.3 Gy fractions by 3D conformal radiation therapy and 21.9 Gy in 10.4 Gy fractions by FSRT.

Our study shows a trend of greater symptomatic responses as well as local and distant control in patients who received higher doses of radiation compared to those who received shorter courses of radiation, although statistical significance was not reached possibly limited by the size of our cohort. Given that these treatments were well tolerated, outcomes may have improved with even higher doses of radiation over 40 Gy as suggested in other series and case (10, 11, 13).

In addition, our study shows that most patients will not experience radiographic regression of their disease. This is consistent both with studies of malignant PCC/PGL, but also with extensive literature on EBRT for benign PGL, which has suggested that this is due to the slow growing nature of these tumors (11). While no target lesion had CR/PR, the three non-target lesions, which had CR or non-CR/non-PR were surgically resected and did completely or partially resolve on imaging. Consistent with other studies, our results indicate that patients may experience long term local and symptomatic control of their disease without significant imaging changes.

It is also important to consider the site of disease being treated when evaluating dose given by EBRT. While many of our patients with bony metastasis responded well to an average dose of 32.6 Gy, a single patient with a primary malignant glomus jugulare experienced PD by RECIST criteria when treated with 32 Gy. Some studies have indicated that these patients may require doses >60 Gy for durable local control (23).

Fewer of our patients were treated with SRS/FSRT and, of these, only one was symptomatic at presentation. As a newer treatment modality, there is less follow-up time for patients with benign PGL treated with SRS although it has shown promising efficacy in many studies. In our study, durable local control was achieved in a greater proportion of patients treated with SRS/FSRT as compared with standard fractionation, although the number treated using this modality was much smaller. It has been suggested, however, that SRS be restricted in use to smaller tumors <3 cm with a more protracted course of radiation either as 3D conformal EBRT or FSRT for larger lesions (23).

Radiation therapy remains a local therapy, and the majority of patients in this study progressed systemically through their radiation treatments, reinforcing the need for concurrent systemic therapy to control distant metastasis. In spite of this, for patients who were able to be evaluated for biochemical responses after radiation there was a decrease in chromogranin A and catecholamine levels, suggesting a decreased overall disease burden after radiation therapy.

As with other reports on the use of EBRT in malignant PCC/PGL, this study is limited by its retrospective nature and the small number of patients treated. However, we have found both 3D conformal EBRT and SRS/FSRT to be effective in controlling symptoms and local progression in patients with both sporadic and familial malignant PCC/PGL. Patients achieved better responses at higher doses in general, although the optimal dose and radiation technique may vary depending on the site treated and size of the lesion. In addition to currently available systemic therapies, EBRT may play a significant role in the control of local disease for these patients.

## Conflict of Interest Statement

The authors declare that the research was conducted in the absence of any commercial or financial relationships that could be construed as a potential conflict of interest.

## Abbreviations

CgA, chromogranin A; CNS, central nervous system; CR, complete response; CT, computed tomography; DP, distant progression; EBRT, external beam radiation therapy; FSRT, fractionated stereotactic radiosurgery; IMRT, intensity modulated radiation therapy; LD, local disease; LP, local progression; MRI, magnetic resonance imaging; NFI, neurofibromatosis type 1; PMN, plasma metanephrine; PCC, pheochromocytoma; PD, progressive disease; PGL, paraganglioma; PGL1, familial paraganglioma syndrome type 1; PGL2, familial paraganglioma syndrome type 2; PGL4, familial paraganglioma syndrome type 4; RECIST, response evaluation criteria in solid tumors; RET, group rearranged during transfection; RTOG, radiation therapy oncology; SD, stable disease; SDHB, succinate dehydrogenase subunit B; SDHC, succinate dehydrogenase subunit C; SDHD, succinate dehydrogenase subunit D; SRS, stereotactic radiosurgery; TTP, time to progression; UMN, urinary metanephrine; VHL, von Hippel–Lindau.
